# Ethical issues in long-term care settings: Care workers’ lived experiences

**DOI:** 10.1177/09697330231191277

**Published:** 2023-08-04

**Authors:** Anna-Liisa Arjama, Riitta Suhonen, Mari Kangasniemi

**Affiliations:** 8058 University of Turku, Finland; 8058 University of Turku, Finland; 60652 Turku University Hospital, Finland; 8058 University of Turku, Finland; The Wellbeing Services County of Satakunta, Finland

**Keywords:** Ethics and dementia care, qualitative research, empirical approaches, care homes, long-term conditions, care of the older person, clinical ethics

## Abstract

**Background:**

Professional care workers face ethical issues in long-term care settings (LTCS) for older adults. They need to be independent and responsible, despite limited resources, a shortage of skilled professionals, global and societal changes, and the negative reputation of LTCS work.

**Research aim:**

Our aim was to describe the care workers’ lived experiences of ethical issues. The findings can be used to gain new perspectives and to guide decision-making to improve the quality of care, occupational well-being and nursing education.

**Research design:**

Focus group interviews were analyzed using a hermeneutic-phenomenological method. The analysis comprised three steps: naïve reading, structural analysis, and comprehensive understanding.

**Participants and research context:**

We randomly sampled LTCS service providers in Finland and 53 care workers with different educational backgrounds from seven organizations participated in focus group interviews in 2021.

**Ethical considerations:**

This was a sensitive study, which was connected to the participants’ individual views of the world, professional ethics and social and health care legislation. The participants' provided informed consent and their anonymity was guaranteed.

**Findings:**

Care workers spoke about their lived experiences of ethical issues in an emotional way, using practical examples. They talked about how they were experts at caring and advocating for residents, balanced the responsibilities of their different roles, and defended their work to the wider society. The care workers said that ethical aspects of their work were too difficult to solve on their own. There were elements of their working environment and practices that caused unnecessary strain and they needed the commitment of managers, organizations, and society to solve ethical issues in LTCS.

**Conclusions:**

Ethical issues were related to the well-being of both residents and care workers and reflected both internal and external pressures. Some issues could not be resolved by individuals and needed input from managers, organizations, and society.

## Introduction

Professional care workers who look after older adults in long-term care settings (LTCS) encounter ethical issues daily. These are related to the challenges they face fulfilling their ethical principles of their professional care duties. Sometimes they face dilemmas or situations where there is no clear course of action,^1^ and they are confused about how to respect the resident’s autonomy,^2,3^ act in their best interests or prevent them from harm.^4–6^ They face several ethical issues when they provide daily care and these can be related to residents’ vulnerability,^7^ their dependency on care workers and the cooperation of family members.^8^ In addition, providing good care can be challenging if there is friction between professionals.^9^

Society expects care workers to ensure that older adults’ rights and their dignity are maintained.^8,10–12^ During the last decade, this has been challenging because of staff shortages and fast-moving changes, such as those caused by the COVID-19 pandemic. This has meant that the care workers have had to meet external demands at the same time as trying to protect residents and ensure they enjoy a good-quality daily life.^9,13–15^

Previous studies on ethical issues in LTCS have focused on providing residents with good care. For example, they have looked at nutritional practices in advanced dementia,^6^ restrictions on moving,^17^ and end-of-life care.^18^ Care workers have reported moral stress when they have tried to make sure residents receive the care they need and this has had a negative impact on working in LTCS. To support care workers in LTCS, we need to understand how they experience the ethical issues connected to their work.^19,20^

## Aim of the study

The aim of this study was to describe care workers’ lived experiences of the ethical issues they faced on a daily basis caring for older adults in LTCS and during the COVID-19 pandemic. These findings could aid decision-making by providing new perspectives on how to improve good care and ethical working in LTC and stimulating ethical conversations about this area of work in nursing education.

## Research methods

We chose the hermeneutic-phenomenological approach, because we were interested in the lived experiences of care workers. This enabled care workers to describe of ethics as subjective and embodied knowledge gained through personal encounters, perceptions, and interactions with the world.^21,22^ Data were collected through semi-structured focus group interviews in Finland in 2021 and we analyzed the data using text-interpretation method based on Ricoeur (1976).^21,22^

### Research environment

The study was carried out in LTCS for older adults. In Finland, this care is organized by public services or procured from private service providers.^23^ Older adults have an equal right to 24-hour care if their functional capacity requires it. At the end of 2019, there were 547,835 people over 75 years of age living in Finland and approximately 17% of them received some kind of services for older adults. Approximately 9% were living in residential care and this equated to 18.4 million care days per year.^23^ According to national regulations, the staffing rate for LTCS in 2021 was 0.6 care workers for each resident.^24^ A total of 30,000 staff were employed in LTCS in 2020. The largest group were licensed practical nurses (LPN) (71%) who have reached level 4 of the European Qualifications Framework (EQF).^25^ The next largest groups were registered nurses (RN) (EQF 6–7) (8%) and care assistants who had no specific degree in social and health care (9%)^23^ In Finland, therapists and those with degrees in social work can also be included in nursing staffing numbers.^24^

### Data collection and recruitment

We collected data by using a semi-structured interview guide through focus groups with care workers. The semi-structured interview method was suitable for this study, because it enabled us to explore themes based on previous research knowledge, but it was also flexible in terms of how the themes were processed and progressed by different groups.^26,27^ We developed the interview guide based on previous literature (Table 1)^28^ and tested this using a pilot interview. Some questions were clarified and the pilot interview was not included in the data.^28^ The interview guide consisted of a warm-up question and two themes.^29^ We chose focus group interviews for data collection method, because we were interested in the information that was produced by the participants and how they discussed ethical issues with each other. It enabled us to understand the participants’ views and experiences of ethical issues.^30^ During the interviews, participants were able to hear, make comments, and share their knowledge.^26^ This study analyzed the key findings, but not the interactions between the participants.

**Table 1.** Themes, pre-defined probing questions, and references used in semi-structured interview guide.

Structure	Themes and references	Probing questions and references
Warm-up question	- How would you describe ethics and ethical issues?	- Specify what makes the situation you described an ethical issue? - How do ethics emerge in your work?
Theme I	- Ethical issues related to the COVID-19 pandemic in daily care ^13,31^	- What should be done: to protect the residents from infection or to allow them to meet relatives^13^? Why?
Theme II	- Ethical issues related to: - Comprehensive care of residents and cooperating with their family members^32-34^ - Being a care worker, being committing to teamwork, and delegating work^35,36^ - Management and the organization you work for^37^ - How LTCS is perceived by society^19,20^	- Specify what makes the situation you describe an ethical issue? - Please provide practical examples from your work - What kind of ethical issues in your work are related to, for example nutrition^16^/restricting movement^17^/recording residents’ data^38^/end-of-life care^18,39^/responding to behavioral symptoms^40-41^/indirect work^13^? - How are challenging situations handled in your workplace? - What kind of sources do you use to follow social debates in your field?
Closing question	- If you could choose, which of the ethical issues related to LTCS should be considered next by our society?	- Specify, what makes the situation you describe an ethical issue? - Is there something you would like to add?

According to the Finnish Institute for Health and Welfare care register,^42^ there were 1,730 service providers at the time of the study and we randomly sampled seven organizations using an electronic randomizer.^43^ One organization was in a metropolitan area, one in a big city, two in average sized cities and three in rural areas in Finland. After we received permissions from the organizations, one researcher (*A-LA*) contacted the nurse managers to help us recruit the participants. The criteria for participation were an employment contract and working in a position that was included in organization’s staffing number. We enrolled 53 care workers in the study (Table 2). The focus group interviews were carried out on the organizations’ premises during the participants’ working time.

**Table 2.** Composition of the 14 focus group interviews.

Level of education/Running number of focus group interviews	1	2	3	4	5	6	7	8	9	10	11	12	13	14	Total
Care assistant with no specific degree in social and health care	1	1									2	2	1		**7**
Licensed practical nurse (EQF 4)	4	3	2		2	1	2	5	2	2	2	2	3	3	**33**
Degree in health care (EQF 6-7)registered nurse, physiotherapist		1	1	2	1	1	2	1		1					**10**
Degree in social work (EQF 6)social work, elderly care professional			2			1									**3**
**Total**	**5**	**5**	**5**	**2**	**3**	**3**	**4**	**6**	**2**	**3**	**4**	**4**	**4**	**3**	**53**

#### Characteristics of the participants

We found that 33 of the 53 participants had vocational level education and had worked in LTCS for more than 10 years. One in 10 participants had taken part in supplementary training after they completed their degree education. (Table 3).

**Table 3.** Characteristics of the 53 participants.

Characteristics	
**Age (years)**
Mean	45
Range	19–74
**Level of education** (%)	
Care assistant with no specific degree in social and health care	13
Licensed practical nurse (EQF 4)	62
Degree in health care (EQF 6-7)	19
Degree in social work (EQF 6)	6
**Service sector** (%)	
Public	69
Private	31
**Years working in older adults’ services** (years)	
Mean	13
Median	11
Range	1–34
**Previous training in ethics** (%)	
None/cannot remember/no answer	30
Only during degree education	60
Supplementary training after degree	10

### Data analysis: Text interpretation

We used the hermeneutic-phenomenological method, based on Ricoeur’s 1976^21,22^ interpretation theory to analyze the data (Table 4). The data were transcribed verbatim and the first phase was naïve reading. We read the text several times to grasp the meaning of the text as a whole and form an initial impression of the content. We formulated the text using phenomenological language, which means that we reinterpreted the content of the text in a way that highlighted the lived experiences of the ethical issues described by the care workers.

**Table 4.** From data collection to text interpretation.^21,22^

Phase	Aim	Implementation
Data collection	To find out how care workers acted in different situations and thereby understand their moral thinking	- Completed 14 semi-structured, focus-group interviews with 53 care workers from 7 organizations - Interviews ranged from 74 to 120 minutes, and the total was 26 hours
Data transcription	To translate the care workers’ comments into text	- Transcribed the text verbatim - Compared the transcribed text to tape recordings and adjusted it - Produced 262 A4 pages, in 11-point Calibri, with 1.5 line spacing
Naive reading	To grasp the meaning of the text as a whole	- Read the text, obtained an overview and formulated it into phenomenological language
Thematic structural analysis	To seek and identify themes to capture the meaning of the lived experiences of the care workers as objectively as possible	- Read the whole text and divided it into meaning units - Read the meaning units and reflected on them against the background of the naïve understanding - Condensed the meaning units and identified the similarities and differences - Condensed all the meaning units with similarities to sub-themes and then assembled them into themes
Comprehensive understanding	To summarize and reflect the themes and sub-themes in relation to the research question, the context of the study and the existing literature	- Read the text as a whole again, keeping in mind the previous phases but remaining open minded and interpreted our findings, together with the literature - Moved back-and-forth between the text and literature during the interpretation to highlight various aspects of the interview text

The next phase was structural analysis (Table 5) where we, according to inductive approach, derived the codes from the data.^44^ Each condensed descriptions about ethical issues were collected equally to uncover diversity and plurality.^21,22^

**Table 5.** Structural analysis. An example of the pathway from direct quotes to themes.

Direct quote	Condensation	Grouping	Sub-theme	Theme
“I need to pee,” said a resident but I wanted to eat my lunch and I knew that the resident had just been to toilet	A resident, who have just visited the toilet, is asking to go to toilet again, but the care worker wants to eat her lunch	Taking care of one's own well-being may sometimes mean ignoring the immediate needs of the resident	Compromise between respecting residents’ autonomy and own well-being	Advocating for residents’ rights for autonomy and proper health care: confronting the ideal and practice
If something extraordinary happens to the resident in the evening, my working day will be longer	The participants prioritize the well-being of the residents and postpone the end of their shift if necessary	In critical situations, the participants are flexible about their own well-being and extends their work shift		

**Figure 1.**
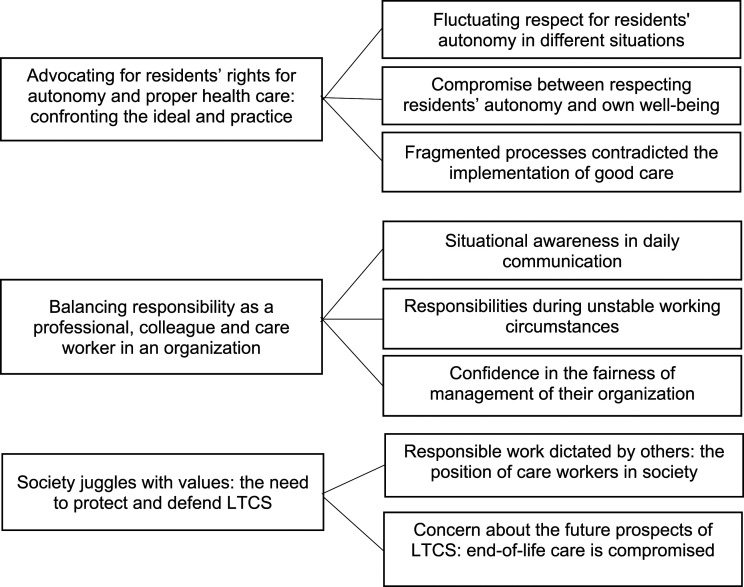
Themes and subthemes based on the structural analysis of ethical issues in the long-term care of older adults, according to the care workers’ lived experiences.

We identified three themes, including seven sub-themes that verified our first impression of the naïve reading (Figure 1). Direct quotes from the participants have been presented in connection with the structural analysis, and these have been identified by the respondent’s role and their length of experience among older adults. The third phase was comprehensive understanding. We summarized and reflected on the themes and sub-themes in relation to the research question and considering the previous literature.^21,22^ This then provided a novel interpretation of the ethical issues in LTCS from the perspective of care workers. The participants brought up ethical issues about the COVID-19 pandemic when they were discussing other things and that is why it has been integrated into those themes. One researcher (*A-LA*) collected the data and the entire working group participated in the analysis.

### Ethical considerations

We stressed the confidentiality of this study because of the ethical nature of the subject. Ethical approval was obtained from the organizations that participated, and written, informed consent was provided by the participants. The focus groups were conducted face-to-face and the participants were informed that all the data would be anonymized and individual participants would not be identified in the results. The author who collected the data (*A-LA*) discussed about authors’ professional background in older adults' services and stressed that the participants were here to discuss their personal views and not those of their organizations. Participants were informed of their right to withdraw their participation without consequences and reassured that only the researchers would have access to the data.^45^

### Findings

In line with the method, we present the naïve reading and structural analysis in the Findings section and the comprehensive understanding in the Discussion section.

#### Naïve reading: The care workers’ need for work-related ethical reflection

The participants talked in an animated and emotional way about the general ethical issues related to LTCS, based on their own daily work and their residents’ best interests. They provided a comprehensive overview of how they justified their choices and discussed how they respected their residents’ self-determination, dignity, and justice in relation to their care. They agreed with the general values of their organizations, but also presented their individual, and sometimes critical, points of view. These included labor shortages in the care sector, which they felt endangered the ethical care of residents.

“*I feel that administration has increased. And we feel we are forgotten. They ask about our opinions, but nothing ever changes.”* (LPN, public sector, worked among older adults 13 years)

#### Structural analysis: Care workers as residents’ advocates with multiple responsibilities in the conflicted LTCS field in society

Participants emphasized the residents’ autonomy and their right to good care and said they had to balance different responsibilities. They also felt the need to protect and defend the role of LTCS in society (Figure 1).

### Theme I. Advocating for residents’ rights for autonomy and proper health care: Confronting ideals and practice

The participants were willing to do whatever it took to advocate for residents’ rights for autonomy and proper health care. However, the ideal situation and the practical implementation of good care did not always match each other.

Participants felt that **respect for the residents’ autonomy fluctuated in different care situations**. This meant that residents’ rights were fundamental for the participants, but they only agreed in principle as they found it challenging to fulfill them during daily care. For example, the residents’ right to make autonomous decisions on minor issues, such as choosing their clothes, was easy to respect and advocate. However, residents’ unhealthy decisions, such as refusing to shower or eat, or having a cigarette, caused confusion because of the tension between health values and the residents’ autonomy. Participants thought it was acceptable to disregard residents’ autonomy if it caused harm to the individual or their social life in the care home.

*‘We discuss the restrictions on resident's rights together. Especially about jumpsuits for some residents. Some employees are of the opinion that they should not be used. But it's usually about messing with secretions, and you must use them.’ (RN, public sector, worked 7 years among older adults)*

The participants said that they needed to **compromise between respecting the residents’ autonomy and their own well-being**. This referred to situations where the participants disregarded the residents’ autonomy to protect themselves, such as ignoring the need to respond to behavioral symptoms by spending time with the resident. Participants respected the right to see family members but were also frustrated when family members took their time and disturbed the privacy of other residents. Participants also felt that they had compromised residents’ autonomy during the COVID-19 pandemic, when they were forced to restrict family visits against the residents’ wishes. At the same time, they said that they felt relief that family members were not present. They felt guilty about that.

*‘They have memory disorders and maybe their perception is different and they don't remember the fact that no-one has visited.’* (RN, public sector, worked 11 years among older adults)

The participants said that good care was compromised because the **care processes in the unit were fragmented**. For example, residents’ daily routines often followed the professionals’ work shifts or were based on catering services. Some of the participants also said that they did not know who the physician in charge was. This meant that residents did not get timely medical help or care. One example of the fragmented processes was when the patient information system did not support continuity of care or recognize the LTCS as the resident’s home.

### Theme II. Balancing responsibilities as a professional, colleague, and care worker in an organization

The participants said they had to balance different professional roles. They had to change from one role to another, suffered from constant work interruptions and were worried about resident safety.

Participants said that these different roles called for **situational awareness during daily communications in changing situations.** For example, it was sometimes challenging to change their communication style when they were dealing with relatives or colleagues and they used inappropriate communication. However, participants knew that there was a risk that family members would misunderstand their humor and well-meaning laughter. Participants were also aware of communication issues related to colleagues’ language skills. They thought that these could lead to situations where the employee did not understand the instructions they were being given and that this could endanger the safety of residents. In addition, working in multicultural work group raised ethical issues about whether work tasks were selected due to care workers’ cultural backgrounds.

*‘A nurse with Muslim background should also take care of the men.’* (RN, public sector, worked 28 years among older adults)

The participants felt confused about their increased responsibilities in the current, **unclear LTCS situations**. They felt that there were tasks that permanent employees were expected to handle. These included medical treatment, guiding students, and handling acute situations. In addition, if the substitute did not have appropriate education or sufficient language skills, the participants had to take on their responsibilities as well as their own. Participants also felt that it was unfair that they were expected to perform roles that were not in their job description, but other professional groups were not.

*‘I am not competent to work as a nurse, but I have a lot to offer as a therapist.’* (Physiotherapist, public sector, work experience 5* *years among older adults)

*‘If I am alone and responsible for 14 residents, I will accept help from any professionals, for example to give residents their breakfast.’* (LPN, public sector, worked over 30* *years among older adults)

Participants said that feeling **confident that managers were fair** was a crucial part of supporting what they felt were ethical issues in LTCS. Nurse managers who were close to care workers, supported them with daily ethical issues, such as organizing residents’ terminal care. However, nurse manager without daily connections to care workers were seen as distant without any understanding of their ethical distress.

*‘The nurse manager is so distant that I prefer to go and talk to a physician.’* (LPN, public sector, worked 20* *years among older adults)

### Theme III. Society juggles with values: the need to protect and defend LTCS

The participants said that their work was based on their ethical responsibility, but they did not have any control over their work they were given. Instead, they felt that they were **dictated to by other people** and they were not able to influence their working conditions. They also felt that LTCS were only valued when family members demanded good care for their loved ones or when employers required care workers to work overtime. They said that they had previously appreciated their permanent employment contracts, but now they felt they restricted their power to influence to their work–life balance.

*‘When you have a permanent job like this, it's a bit like a prison sentence. If something unexpected happens in life and you need a vacation day or you want to change shifts or days off with someone, then no dice.’* (LPN, public sector, worked 17* *years among older adults)

Participants were surprised about the attention that the media paid to the ethical issues around their work and LTCS during the COVID-19 pandemic. However, they felt that they were left alone with their ethical problems and were not defended in society when they were on the front line caring for residents with COVID-19. Instead, the media blamed care workers for spreading the virus, even when they agreed to be vaccinated for ethical reasons, just to protect residents.

According to the participants, the reality did not match what was expected from good care and the conditions in LTCS often contravened ethical principles. There was no time to spend with residents and they often worked in restless environments, because they did not have the resources to respond to residents’ behavioral symptoms.

*‘We just go to the door to see if the resident is already dead and that’s the only terminal care we can provide.’* (LPN, public sector, worked 17* *years among older adults)

Participants expressed their **concerns about the future ability** of LTCS settings to tackle ethical issues, as they thought that care in the residents’ later years was compromised. They were afraid that values were being eroded, with older adults being exploited by both family members and service providers who wanted to maximize their profits. The participants said that LTCS work was not attractive for future generations. The education that new generations of staff received was seen as insufficient, desperate and unethical due to workforce shortages. For example, sometimes nursing students passed their training, even though the care worker who was supervising them thought that their skills did not meet the requirements of the curriculum. The participants also thought that there were some ethical issues about how the authorities monitored public and private service providers and that both kinds of organizations should be subjected to the same level of scrutiny. However, they suspected that the methods that were being used to do this were complicated and time-consuming in relation to the benefits they achieved. In addition, the participants felt that it was unethical that care workers employed by some organizations had been instructed to falsify information about the residents’ conditions and the number of staff to the authorities.

*‘There is no shortage of trained nurses in Finland. After all, there are some 70,000 trained nurses who are working in another field.’* (LPN, private sector, worked 25* *years among older adults)

*‘I hope that residents’ family members will complain about the circumstances to the authorities.’* (RN, public sector, worked 20 years among older adults)

### Discussion: Comprehensive understanding

This study produced an understanding of the ethical issues involved LTCS from the care workers’ perspectives. The topical areas that were identified were advocating for residents and having multiple responsibilities in the conflicted LTCS field in society. Working conditions^9,14,15^ and staff shortages highlight the need for urgent recognition of ethical issues around safeguarding the nursing care provided by competent professionals. Ethical issues have rarely been studied from the perspective of care workers and this study addresses that gap in the literature.

This study showed that care workers faced daily, ongoing ethical issues in LTCS. They were complex and included numerous of stakeholders, such as family members and different professional groups. Due to their education and work experience, the care workers were the experts when it came to the content of residential care, but the ethical issues were too broad for them to handle by themselves. This requires ongoing reflection on the ethical issues, with support from organizations and society.

The care workers were committed to advocating for their residents’ autonomy and providing kind and respectful care for older adults who depended on help from others.^9,14^ Workforce pressures could jeopardize the good care provided if care workers felt that they had more responsibilities than resources, and they started to work mechanically and had difficulties in prioritizing work tasks.^47^ The care workers emphasized that it was important that advocating for residents did not compromise their own well-being. Our data agreed with other studies,^1,9,15^ that it was possible that a care worker could be the only educated professional on a shift, assisted by deputies with no formal education. If these situations were not reflected in their organization, this could lead to a slippery slope, when it came to ethics. This refers to a phenomenon where small mistakes are initially ignored and gradually grow into harmful practices. Unraveling situations created by conflicting values and getting feedback on them, is a prerequisite for avoiding this issue.^10,36^ Previous literature also described moral distress, which arose from external obstacles that prevent employees from acting in accordance with their ethical principles. Moral distress has been associated with decreased job satisfaction, increased burnout, and staff expressing their intention to leave their jobs.^14^ It has been estimated that the prevalence of moral distress is nearly twice as high in caring for older adults than in other health and social care services work.^37,46^

The lived experience of care workers in this study reflected their interpersonal orientation regarding LTCS ethics. It is noteworthy that participants highlighted the importance of maintaining relationships between people and identified emotional registers^48,49^ when describing ethical issues. In addition, they focused on their relationships with the older people being cared for. According to the ethics of care, the care worker guides the activity until both parties can spontaneously interact with each other. However, implementing the ethics of care may be a burden if the care worker is not supported.^50-52^ The attitudes towards family members varied. Previous studies have shown that families can have an impact on care workers’ decision making and cause moral distress.^14,15,46^ This was accentuated during the COVID-19 pandemic.^31^

Care workers have multiple responsibilities. Ethical issues arise from acting in different roles and momentary judgment changes in different situations.^10^ Their feeling about being responsible strongly follows the need to do the right thing in every situation.^52^ Care workers feel that they are both morally and criminally responsible for their actions,^6^ even when situations are beyond their control. If there are constant interruptions or staff changes then responsibility breaks down and it may slow them down—or even prevent—them from establishing positive practices. This can reduce resident -safety and the staff member getting to know residents.^53^ This study emphasizes how the care workers’ lived experiences of ethical issues were inherently connected to their working community and colleagues and reflected the views of the group. The care workers referred to, and pleaded for, shared and agreed care values. Adhering to shared values has been found to be a prerequisite for successful work, but risks can arise if care workers obey agreed rules instead of doing what is best for resident. It can be a major obstacle for care workers to implement appropriate ethical actions if creativity and critical reflection are lacking.^5^

Care workers said that negative comments from society about caring for older adults made the field less attractive. They said that LTCS would be more attractive if they received a reasonable salary for their work, had working conditions that enabled them to focus on caring for residents and had familiar colleagues around them. Care workers emphasized the importance of having a supervisor present who could support them in their daily work. Studies have reported that supervisors represented, and built trust, in the management of organization.^54^ Their presence was considered important for establishing an ethical climate in LTCS, which was associated with high-quality and individualized care.^46^ Our data reinforced the notion that major changes are occurring in LTCS. One visible change for care workers was teams with different cultural and educational backgrounds. Care workers’ professional and language skills are regulated by law^24^ in Finland, but compliance was insufficient and care workers described situations where this endangered resident safety. In addition, the societal role of care workers training future professionals also caused conflicting feelings. The care workers felt they did not have enough time to train students, although it is known that a successful training requires planning and resources for guidance.^55^ Care workers can only be expected to develop and maintain their competence through continuous education if they are supported by their supervisors and managers.^56^ Only 10% of care workers in our study had participated in supplementary ethics training during their working career.

### Strength and limitations

Ricoeur’s method of text interpretation was effective for conceptualizing the most fundamental ethical issues in caring for older adults in LTCS. We collected the data in the fall of 2021, when the COVID-19 pandemic was affecting society and long-term care units. Carrying out interviews and using critical interpretation to analyze them, enabled us to understand the lived experiences of the care workers and provided new insights.^21^

We selected the organizations randomly and conducted the interviews face-to-face, despite the long distances involved. Because the research topic was complex, and previous knowledge was scarce, focus groups were a suitable method for collecting data.^57^ All employees were offered the opportunity to participate, but it seems that only employees who spoke Finnish well took part in the focus group interviews. The data were collected until no new themes emerged from the discussions.

The phenomenological hermeneutical method that we used has been shown to provide a clear and practical process, which is well suited to addressing ethical issues.^22^ This method also enables researchers to interpret text in different ways.^21,22^ All authors participated in completing and conforming the analysis to mitigate any one-sided text interpretation.

## Conclusion

This study was based on focus group interviews with care workers looking after older adults in LTCS. It generated various ethical issues around LTCS, which were related to professional care workers in organizations. The ethical issues in this area of care are so broad that they cannot be resolved just by the care workers themselves. There are issues in the operating environment and practices that cause unnecessary strain on care workers. The ethical issues that were discussed were related to the well-being of both residents and care workers. Responding to requirements of residents, their family, colleagues, and organizations is essential for providing high-quality care and ethical standards. Organizations, managers, and society need to get involved in how care workers support older adults in LTCS services and ethical issues play a key role in this process.
